# Smart Force Sensing in Robot Surgery Utilising the Back Electromotive Force

**DOI:** 10.3390/s25030777

**Published:** 2025-01-28

**Authors:** Storm Chabot, Koen Schouten, Bart Van Straten, Stefano Pomati, Andres Hunt, Jenny Dankelman, Tim Horeman

**Affiliations:** 1 Department of BioMechanical Engineering, Delft University of Technology, 2628 CD Delft, The Netherlandsk.schouten-1@tudelft.nl (K.S.); b.j.vanstraten@tudelft.nl (B.V.S.);; 2 Asensus Surgical, Durham, NC 27703, USA; 3 Department of Precision and Microsystems Engineering, Delft University of Technology, 2628 CD Delft, The Netherlands

**Keywords:** robotic surgery, laparoscopy, smart force sensing, back electromotive force, force feedback

## Abstract

Since the introduction of robot-assisted laparoscopic surgery, efforts have been made to incorporate force sensing technologies to monitor critical components and to provide force feedback. The advanced laparoscopic robotic system (AdLap RS) is a robotic platform that aims to make robot technology more sustainable through the use of the fully reusable shaft-actuated tip-articulating (SATA) instruments. The SATA instrument driver features electronics and sensors exposed to the sterile environment, which complicate the sterilisation process. The aim of this study was to develop and validate smart sensing in stepper motors using the back electromotive force in a newly developed Smart SATA Driver (SSD), eliminating the need for sensors in the sterile environment. Methods: The stepper drivers were equipped with TMC2209 ICs featuring StallGuard technology to measure back EMF. The tip was actuated up until a set StallGuard threshold value was reached, at which the resulting tip force was measured. This cycle was repeated ten times for a range of threshold levels. A regression analysis with a power series model was used to determine the quality of the fit. Results: The SSD is capable of exerting tip forces between 2.4 and 8.2 N. The back EMF force test demonstrated a strong correlation between obtained StallGuard values and measured tip forces. The regression analysis showed an R-squared of 0.95 and a root Mean squared error of 0.4 N. Discussion: The back EMF force test shows promise for force feedback, but its accuracy limits real-time use due to back EMF fluctuations. Future improvements in motor stability and refining the back EMF model are needed to enable real-time feedback. Conclusion: The strong correlation during the back EMF force test shows its potential as a low-budget method for detecting motor stalls and estimating tool–tissue forces without the need for sensors in laparoscopic instruments.

## 1. Introduction

In the twenty-first century, minimally invasive surgery has replaced open surgery as the preferred surgical approach [1]. More recently, robot-assisted surgery (RAS) has entered the surgical field. This approach involves robotic arms on which long and slender laparoscopic instruments are mounted that enter the abdominal cavity through a small incision. The surgeon controls the robotic arms and laparoscopic instruments (slave) from a console system (master). Studies suggest that surgeons can perform procedures with greater precision, accuracy, and ergonomics using master–slave robotic systems [2]. Some systems provide an immersive 3D view and enhanced dexterity through features such as tremor filtering, motion scaling, and instrument articulation [3,4,5]. These systems are based on various combinations of actuation and force and motion sensor technology.

The first well-known robotic system, the Da Vinci robot, was introduced by Intuitive Surgery in 1999 [6]. Since then, the field of robotic surgery has developed rapidly, with over 900 new robotic surgical platforms being installed worldwide each year [7]. Currently, most of Intuitive Surgery’s patents are expiring, creating opportunities for new surgical robot developers [8].

Although the development, adoption, and use of robotic surgical systems are growing rapidly, robotic laparoscopic surgery also brings new challenges. Among many robotic surgical platforms, two issues are common: the considerable expense associated with robotic surgery and its unsustainable character [9]. Notably, these issues are highly correlated.

To address disposable waste in robotic surgery, a Dutch-Italian-Spanish initiative aims to develop alternative technologies that make future surgical robots more sustainable, more affordable, and more accessible in less wealthy regions. The advanced laparoscopic robotic system (AdLap RS) is the first waste-free robotic surgical platform that is currently being developed by the initiative [10].

**Figure 1:**
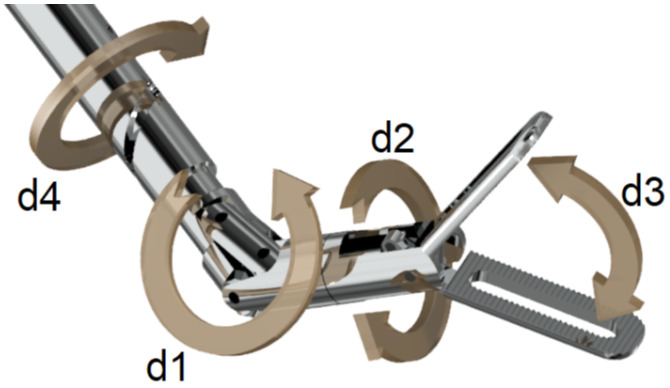
The four degrees of freedom (d1–d4) of the SATA instrument [11].

**Figure 2:**
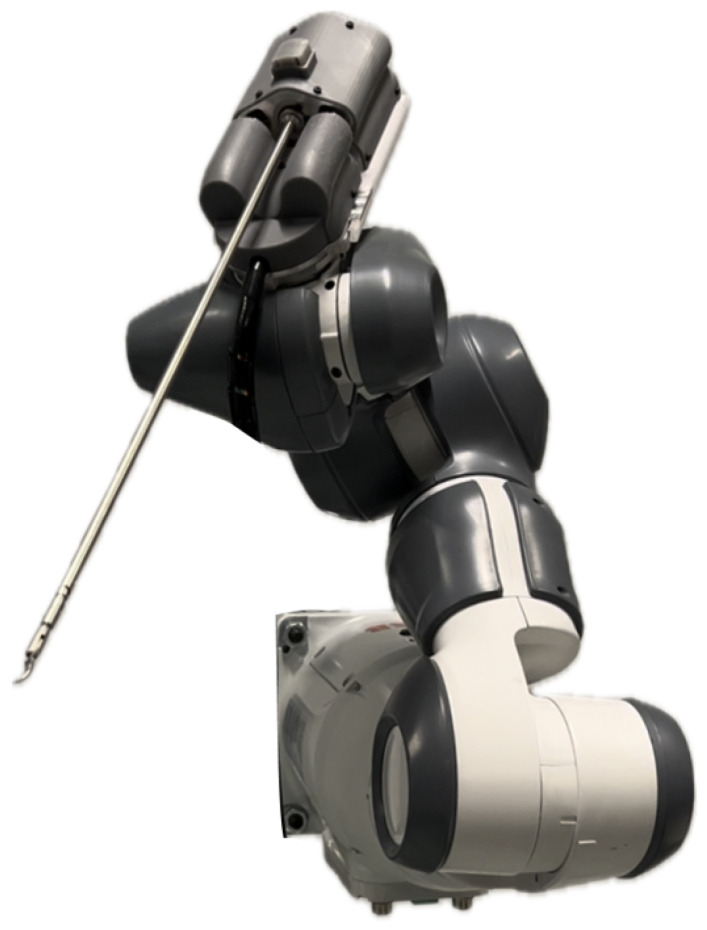
The Smart SATA Driver (SSD) of the AdLap RS. The SSD with the SATA instrument is connected to the YuMi IRB14050 robotic arm (IRB14050, YuMi, ABB, Auburn Hills, MI, USA).

The robotic platform is envisioned to be compatible with the shaft-actuated tip-articulating (SATA) instrument (Figure 1). The SATA instrument was developed as an alternative to current cable-driven instruments and provides a reusable, modular, and sterilisable solution [11]. The end-effector in the SATA instrument is articulated by rotating hollow shafts rather than cables, eliminating cable wear. The design principles used in developing this instrument also apply to the AdLap RS: an instinctive, maintenance-friendly, and modular design philosophy using a ‘bare-minimum design’ approach [11]. An affordable and sustainable robotic driver system was developed to robotically manipulate the four degrees of freedom (DOFs) of the SATA instrument (Figure 1). This driver is reusable, featuring modular components that can be inspected and cleaned, and uses the same instrument shafts and tips as the handheld version of the SATA instrument (Figure 1 and Figure 2).

Different from their predecessor, the modular interfaced sterilisable laparoscopic instrument drive (MISLI drive) [12], the new AdLap drivers have no electronics and sensors at the gearbox side, which is exposed to the sterile environment, to prevent complicating the sterilisation process.

Using electronics in a hospital’s Central Services and Sterilisation Department (CSSD) often conflicts with local guidelines. Reusable instruments undergo cleaning, disinfection, and sterilisation at high temperatures (up to 134 °C) and with chemical agents. These harsh conditions can damage electronics through corrosion, short circuits, and other failures. To avoid this, either specialised electronics must be designed, or the use of electronics must be omitted [13].

To operate the SATA instrument safely and to provide a form of instrument force feedback, information can be derived from the actuators by linking the instrument and actuator loading to the power consumption.

Measuring the back electromotive force (back EMF) generated in the motor could eliminate the need for sensors. During motor actuation, the rotor induces a current in the stators, known as back EMF. By measuring back EMF, detecting motor stalls and determining motor load should be possible. This technique can be used for calibration purposes. Additionally, force limitation or force feedback could be achieved by monitoring motor load via back EMF measurements during actuation. Utilising back EMF for monitoring forces has not been previously explored for robotic surgery. Some commercially available stepper drivers have built-in back EMF sensing technology, making this an attractive option. The TMC2209 stepper motor drivers (TMC2209, ADI Trimanic, TRINAMIC Motion Control GmbH & Co. KG, Hamburg, Germany) convert back EMF measurements into so called “StallGuard” values, which are used to detect when the motor stalls [13]. The goal of this study is to develop and validate smart sensing using the back EMF in the newly developed SSD and to investigate whether it is capable of sensing reaction and grasping forces after calibration without requiring electronic components in the sterile environment or any other additional electronic components.

## 2. Materials and Methods

### 2.1. Smart Sensing

The Smart SATA Driver (SSD) will be equipped with (smart) sensing capabilities to ensure the safe operation of the SATA instruments. Since SATA instruments have a limited range of motion in three out of four DOFs, detecting their allowable respective ranges prevents damage to the instruments due to overactuation. Sensors and electronics, however, should be kept outside the sterile environment to overcome potential damage and corrosion issues as a result of CSSD steam sterilisation procedures.

The forces acting on the motor can be indirectly measured by monitoring the back EMF, using the embedded measurement feature available in certain commercial stepper motor drivers [13]. This approach enables force measurements without additional components, aligning with design requirements for simplicity, low complexity, and full sterilisability [14]. StallGuard technology, developed by Trinamic and integrated into some stepper drivers, uses back EMF measurements to provide StallGuard values, which can be used to estimate motor load and detect stalls [13]. These stepper drivers are readily available and straightforward to use due to the serial communication protocol, making them the prime candidate to use.

#### 2.1.1. Back EMF Background

According to Faraday’s Law, a voltage is induced in a conductor when it is exposed to an alternating magnetic field. This phenomenon is known as back EMF [15].

During motor actuation, the rotor spins, causing the stator coils (conductors) to experience an alternating magnetic field. Consequently, a voltage is induced in the coils due to this alternating magnetic field. This induced voltage, denoted by ε, opposes the supplied voltage from the stepper driver and can be calculated using Faraday’s Law Equation (1),

(1) $$\varepsilon =-N\frac{\Delta \phi }{\Delta t}$$

where ϕ represents magnetic flux, *N* the number of coil windings, and *t* the time. The magnetic flux can be calculated by Equation (2),

(2) ϕ=B·A·cos(θ)

where *B* represents the magnetic field, *A* is the surface area the magnetic field passes, and θ is the angle between the surface and the magnetic field lines. θ is a function of time and angular velocity ω,

(3) θ=ω·t

Based on Equations (2) and (3), it can be derived that the change in magnetic flux can be expressed as:

(4) $$\frac{\Delta \phi }{\Delta t}=-B\cdot A\cdot \omega \cdot sin(\omega t)$$

taking motor characteristics into consideration (*C*), the generated back EMF can be calculated by combining Equations (1) and (4).

(5) $$\varepsilon_{emf}=C\cdot \omega \cdot sin(\omega t)$$

where *C* incorporates constants such as the magnetic field strength of the rotors (*B*), the surface area (*A*), the number of coils (*N*), and the motor characteristics.

**Figure 3:**
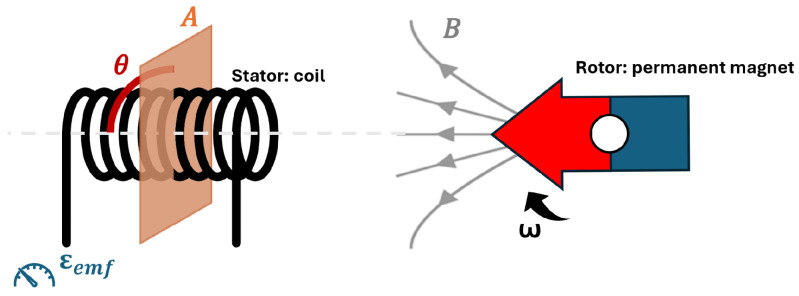
Schematic overview of the stator (coil) and rotor (permanent magnet).

Thus, the back EMF is proportional to the change in magnetic flux Equation (1), which depends on the angular velocity Equation (4). Based on Equation (5), the back EMF is periodic and proportional to the angular velocity of the motor rotor [16,17] (Figure 3). Therefore, with a higher angular velocity (ω), more back EMF is generated as the magnetic flux changes at a higher rate. No back EMF is generated when the motor is not actuated and ω is zero. Similarly, when motor stall occurs, back EMF will be zero, as the angular velocity and thus the change in magnetic flux become zero. Therefore, if stepping pulses are sent to the motor but no back EMF is detected, it indicates a motor stall [16,17,18].

**Figure 4:**
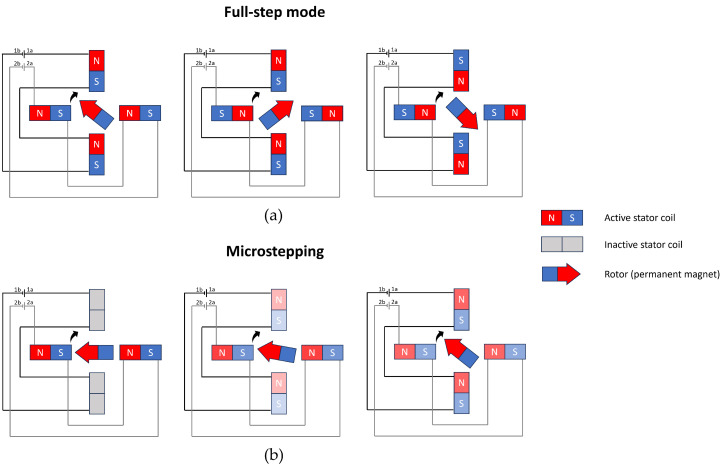
Common actuation sequences for stepper motors. (**a**) Stepper motor rotating in full-step mode. The coils are always energised, which generates high motor torque; (**b**) stepper motor rotating in microstepping mode. High position resolution can be achieved by dynamically changing the current in the coils at the cost of torque loss.

For measuring back EMF, microstepping is required (Figure 4b). In full-step mode (Figure 4a), the motor moves discretely from one position to another, causing a rapid rise and fall in angular velocity, leading to momentary pauses in motion. Consequently, according to the formula for back EMF Equation (5), the back EMF during these instances becomes zero. This poses a challenge as discrete stepping can no longer be distinguished from motor stall. Moreover, in full-step mode, stepper motors operate with all coils energised simultaneously. This complicates the accurate measurement of back EMF, as the supply voltage dominates over the back EMF signal.

In microstepping mode, the current in each coil follows a waveform pattern with polarity shifts. These shifts create brief moments, known as zero-current crossings, when a coil is not energised by the stepper driver. During these intervals, the rotor continues to rotate due to the other actively energised coils. Hence, these (inactive) coil(s) are exposed to an alternating magnetic flux from the rotor, and, according to Equation (5), back EMF is generated. During the zero-current crossings, the generated back EMF can be measured accurately [18]. In the illustration of Figure 4b, back EMF can be measured over coils 1a and 1b in the first phase since these coils are not energised by the stepper driver at that specific instance.

#### 2.1.2. Estimating Load Angle to Motor Load

**Figure 5:**
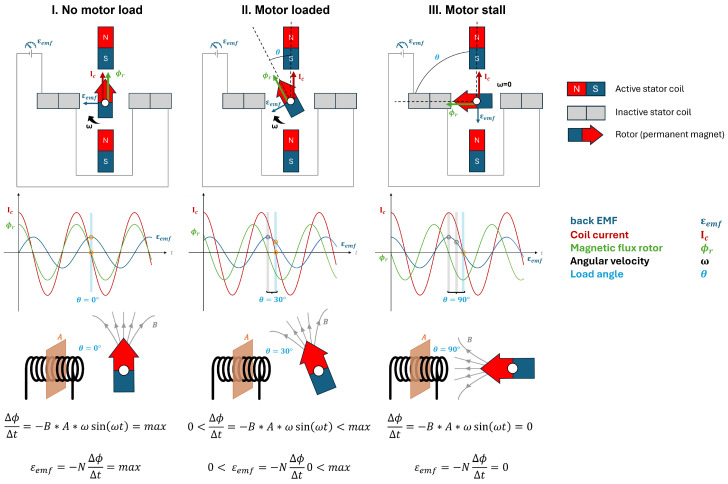
Simplified representation of measuring back EMF during zero-current crossing without any motor load (**I**), while motor experiences load (**II**), and while motor stalls (**III**).

Motor stall and motor load are determined based on the back EMF measurements during zero-current crossings. This involves complex calculations to estimate the load angle (θ). The load angle is the angle between the rotor’s magnetic vector field and the stator magnetic vector field (i.e., the stator current vector) [16,17,18]. To understand how the load angle is determined, three scenarios should be considered:

- No motor load: the motor operates without any load, resulting in a load angle of zero (Figure 5I). The magnetic field lines of the rotor and stator align perfectly, yielding a load angle of zero. This corresponds to a maximum back EMF value proportional to the angular velocity ω.
- Motor loaded: The motor experiences a load, resulting in a load angle greater than zero but less than 90 degrees (Figure 5II). The rotor begins to lag behind the stator’s magnetic field. The load angle increases and the angular velocity stays constant; however, there occurs a phase shift and therefore the back EMF decreases.
- Motor stall: the motor load is too high, causing the motor to stall and skip steps. In this case, the load angle is 90 degrees (Figure 5III). The back EMF diminishes, as the load angle becomes 90 degrees and the angular velocity zero.

Thus, measuring back EMF during zero-current crossings can be used to determine motor load [18].

#### 2.1.3. StallGuard in Practise

**Figure 6:**
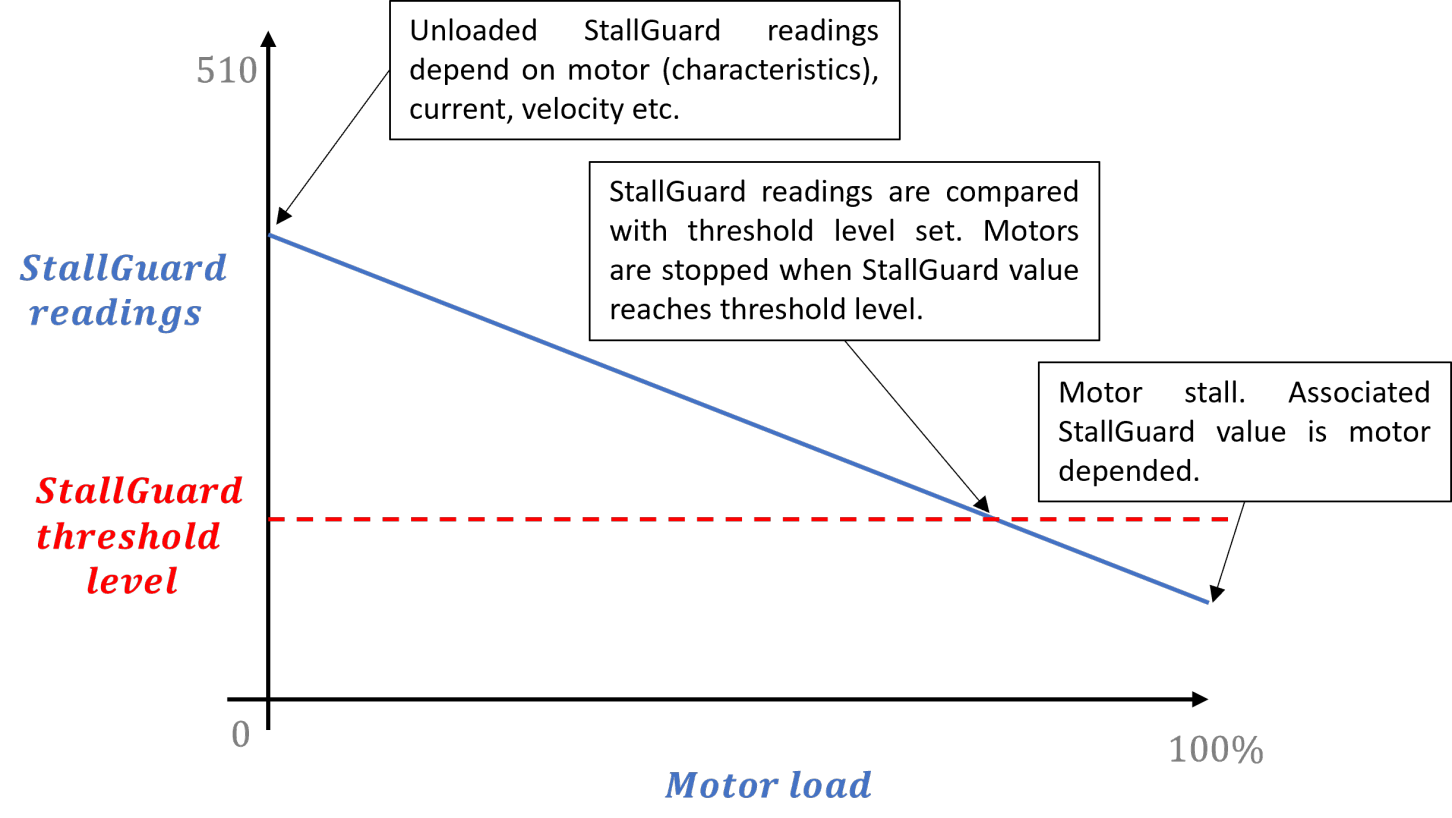
Interpretation of StallGuard values and motor load.

Additional calculations are required to cancel out noise and provide useful feedback on motor load based on back EMF measurements and the stator’s current vector. The TMC2209 stepper drivers are equipped with StallGuard4 technology, which measures the back EMF generated during these zero-current crossings and translates it to an interpretable StallGuard value [13]. These StallGuard values can be used to detect motor stall and determine motor load, as schematically indicated in Figure 6. The back EMF and thus the StallGuard values highly depend on motor characteristics, the rotor’s permanent magnet, the speed, current, voltage, coil windings, and microstep settings.

### 2.2. Experimental Validation

Experiments were conducted to calibrate the tip force sensing, determine the correlation between stepper motor speed and StallGuard data, and interpret StallGuard data to obtain reaction grasping forces.

#### 2.2.1. Tip Sensor Calibration

To calibrate the tip sensor, a FUTEK load cell (FSH00095/FSH01888) (Irvine, CA, USA) was mounted on a linear stage (PI stage) [19]. A force curve was applied to the tip sensor using a second exponential fit analysis by reading out the force–displacement measurements of the load cell. The tip sensor’s output (voltage) was captured using Coolterm. The voltage data (from the tip sensor) and the force data (from the load sensor on the linear stage) were processed in Matlab. A voltage–force relationship was established by combining the tip sensor’s voltage data with the force readings from the load cell on the linear stage.

#### 2.2.2. Back EMF Force Test

Using TMC2209 stepper drivers, a StallGuard value based on the measured back EMF during zero-current crossings in a stepper motor can be obtained. This test measures whether different StallGuard threshold levels can be set to limit the tip closing force.

**Figure 7:**
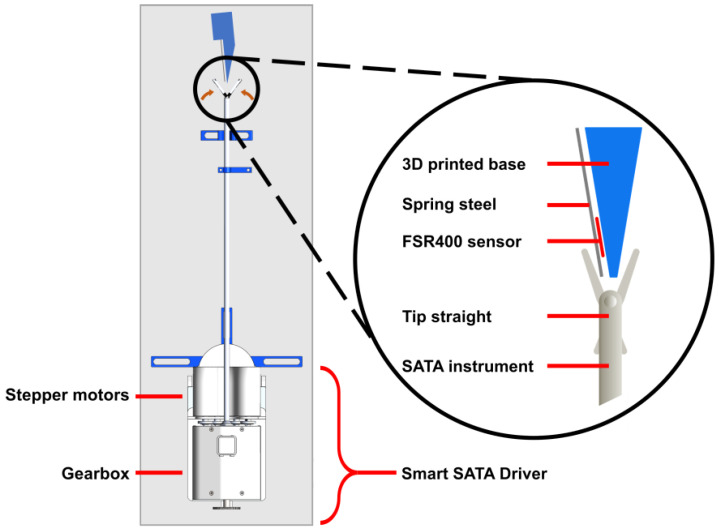
Illustration of the end-effector closing force test setup. The Smart SATA Driver was mounted on a Thorlabs breadboard and the SATA instrument was inserted and coupled in the Smart SATA Driver. A FSR400 force sensing resistor was positioned in the opened end-effector.

During these tests, an FSR400 force-sensing resistor from Interlink Electronics (Camarillo, CA, USA) was used to measure the end-effector closing force. This sensor was mounted in a 3D-printed PETG housing under a 0.2 mm thick steel blade to distribute the clamping force evenly and avoid local deformations of the soft force sensor (Figure 7).

A C++ script was written to control the opening and closing of the end-effector. During end-effector actuation, StallGuard values were continuously monitored. Actuation ceased automatically once the StallGuard values obtained from the stepper driver reached a pre-defined threshold level.

The threshold value range was determined by measuring the StallGuard value during unobstructed actuation up to full end-effector closure. For each StallGuard threshold value set, the force on the tip sensor was constantly measured while the close tip button was pressed. The speed of the motor driving the end-effector, known as the transporter motor, was configured to 21.45 revolutions per minute (rpm), which corresponds to a tip closing velocity of 5.5 degrees per second. This rotational motion is then converted into a translation motion within the gearbox module of the SSD.

The close-tip button is held until the motors are automatically stopped, which happens as soon as the (averaged) StallGuard values obtained from the stepper drivers reach the threshold value set. Hereafter, the close-tip button is released, and the open-tip button is pressed and held until the tips of the end-effector are completely separated from the tip sensor. This cycle was repeated ten times per threshold level set.

The tip sensor measured the end-effectors’ closing forces at a frequency of 100 Hz. These data were captured using Coolterm and stored in CSV files. The data was imported to and processed with Matlab [20,21]. For each test condition (TC), the maximum forces were identified and the average force was calculated. Boxplots were generated to provide an overview of the results. A regression analysis using the second-order power series model was employed to investigate the relationship between the threshold levels and the measured tip forces. This model, implemented using Matlab’s fit function, allowed for a curve fitting with Equation (6).

(6) $$f\left(x\right)=a\cdot x^{b}+c$$

The variable *x* represents the StallGuard threshold levels and f(x) represents the corresponding tip force. The coefficient of determination ($R^{2}$) was calculated to evaluate how accurately the model estimates the tip forces at different StallGuard (threshold) levels [22,23]. The coefficient of determination is a statistical method to measure the relationship between two variables: the independent variable (StallGuard threshold level) and the dependent variable (tip force).

## 3. Results

**Figure 8:**
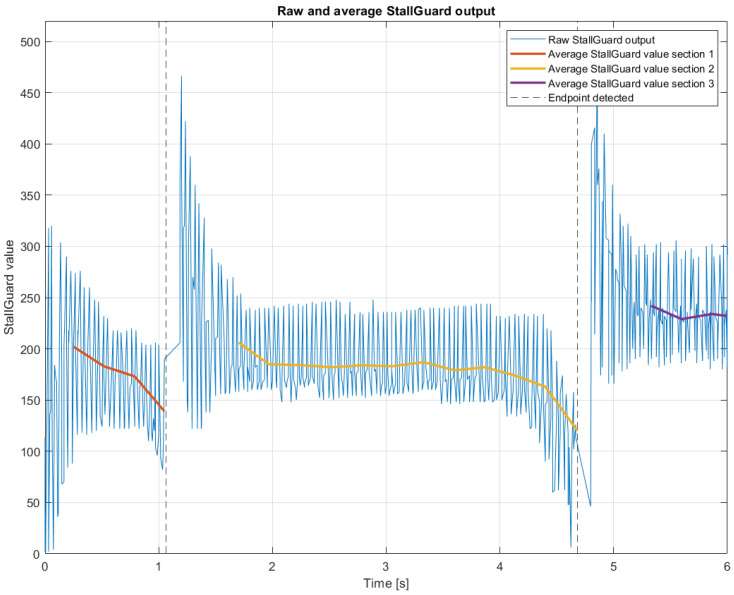
A plot of the StallGuard values and averaged StallGuard values obtained during the calibration cycle of the SSD and instrument. During this cycle, the range of motion for instrument bending is identified.

StallGuard technology was implemented in the SSD using TMC2209 stepper drivers. During the instrument calibration sequence (Figure 8), the SSD automatically identifies the range of motion of tip bending and end-effector closing based on StallGuard data. Subsequently, the instrument returns to a home position (tip straight and end-effector closed), after which the instrument can be actuated within safe margins of the identified range of motions.

Furthermore, the force with which the end-effector is closed (within the identified range of motion) is continuously monitored using StallGuard. If the StallGuard values exceed a predefined threshold, the closing actuation stops (regardless of whether the close tip button is still pressed) to limit the end-effector closing force.

### 3.1. Tip Sensor Calibration

**Figure 9:**
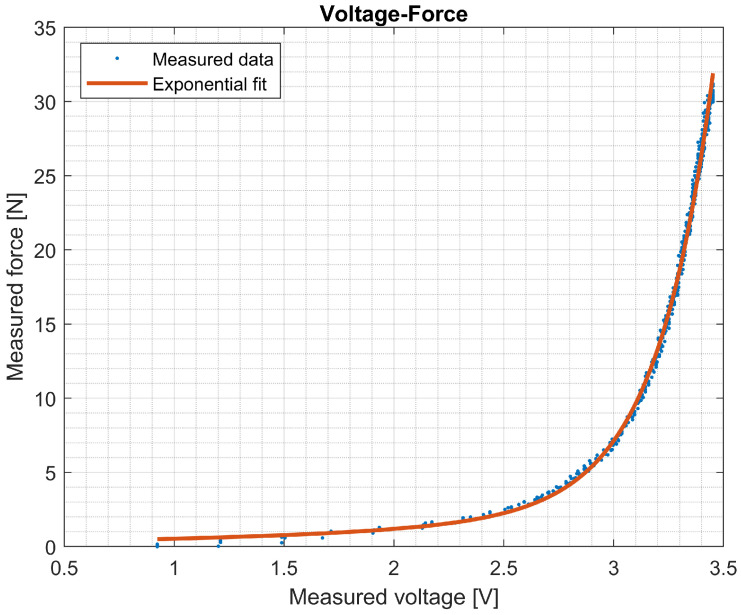
Calibration data for tip sensor. The voltage readings from the tip sensor are plotted against the force measurements from the load cell mounted on the linear stage. An exponential relationship was established to calibrate the tip sensor.

**Figure 10:**
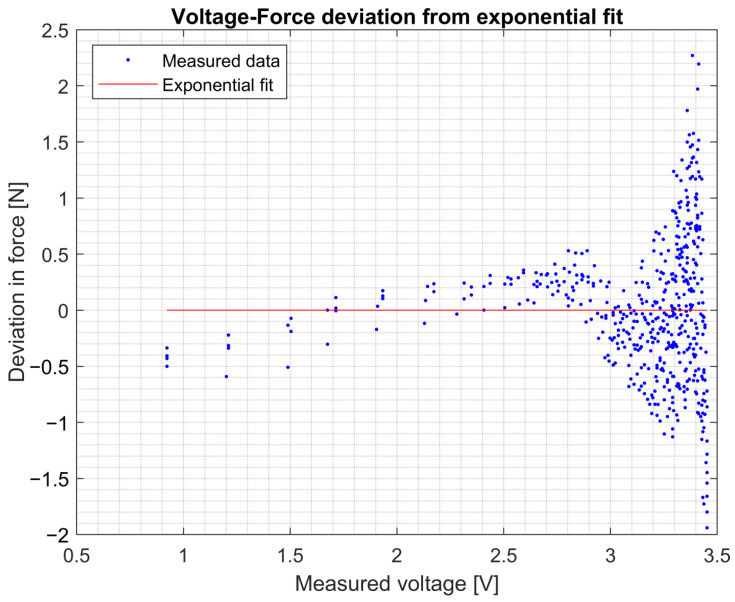
For each data point, the deviation between the fitted curve and the corresponding force is illustrated. The model’s accuracy for the tip sensor is approximately 0.5 N within the voltage range of 1 to 3 V. Beyond this threshold, the accuracy of the measurements diminishes.

The voltage data of the tip sensor and the force readings from the load cell on the linear stage resulted in the voltage–force relationship shown in Figure 9. The second exponential fit analysis resulted in Table 1. This exponential curve was used to translate the voltage readings obtained by the tip sensor into force measurements. As can be derived from Figure 9 and Figure 10, the sensor has a baseline offset. This offset does not affect the non-zero force measurements when force is applied to the sensor. Using this setup, the sensor can accurately measure up to 10 N. Accuracy diminishes for higher forces up to 35 N.

### 3.2. Back EMF Force Test

The StallGuard value obtained from the stepper driver during unobstructed actuation was 240. Therefore, the initial threshold level was set at 230 (high stall sensitivity). The StallGuard value, obtained when the end-effector was fully closed (and the motor started to stall), yielded 130 (low stall sensitivity). Threshold values were decremented from the initial StallGuard value, 230, at intervals of 10, to the lowest StallGuard value observed, 130. This resulted in eleven TCs as, listed in Table 2.

**Figure 11:**
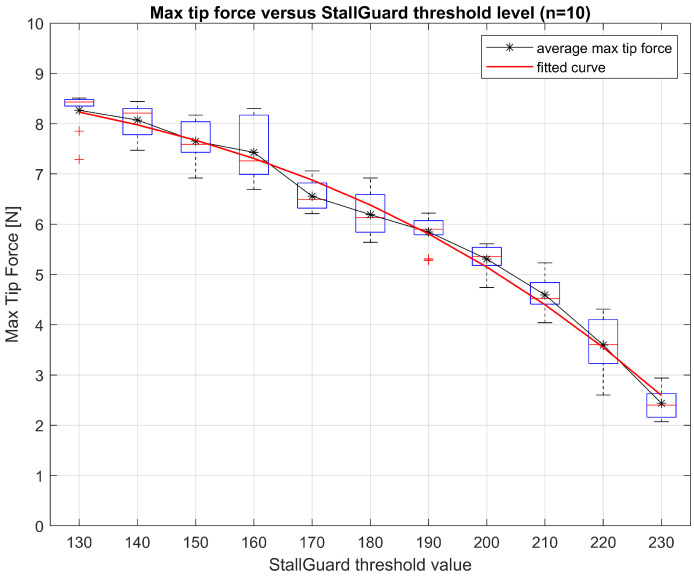
Boxplots of maximum tip force measured at different StallGuard threshold levels between 130 and 230. A regression analysis was performed using the second-order power series and is plotted as the fitted curve.

The boxplots in Figure 11 show the distribution of the maximum tip force per threshold level. Two outliers were observed at StallGuard threshold level 130 (TC1) and 190 (TC5). The average tip forces, measured when motor actuation was ceased because the StallGuard threshold level was reached, are listed in Table 2.

The regression analysis using the second-order power series model resulted in Table 3 and Figure 11.

The coefficient of determination, $R^{2}$, was calculated to be 0.95 (in a 0–1 range). The $R^{2}$ represents how well the model fits the observed outcomes [24]. The root mean squared error (RMSE) of the model was calculated at 0.4 N. A summary of all relevant experimental calibration and testing results can be found in Supplemental File S1.

## 4. Discussion

StallGuard technology was integrated into the SSD, allowing for motor load and motor stall detection by measuring the back EMF without having electronics or sensors in the gearbox. This technology facilitated the development of an automatic instrument calibration feature and served as a force-limiting feature for the end-effector of the SATA instrument. The back EMF force test demonstrated that StallGuard could be used for force feedback purposes.

Utilising the back EMF generated in the motor for motor load and stall detection eliminates the need for additional electronic components in the sterile environment or any other additional electronic components. Therefore, this method has the potential to to be used in the development of lighter and more resilient instrument drivers that can be autoclaved without expensive en sensitive sensors that can limit the lifespan. This is a step towards a future of more sustainable, more affordable, and more accessible surgical robots, especially for low-to-middle-income countries.

### 4.1. Back EMF Force Test

The back EMF force experiments demonstrate a strong relationship between obtained StallGuard values and measured tip forces. The regression analysis using the power series model yielded a coefficient of determination (R-Squared) of 0.95 and an RMSE of 0.4 N. The second-order power series seems to capture the trend of the data closely enough and with enough accuracy to recognise the most important changes in force. The model’s accuracy is reliable for applications such as force threshold limitations or calibration purposes that are relevant for repositioning of the instrument in the trocar or preventing excessive force on the abdominal incision. However, the model’s accuracy is less reliable for real-time force feedback in relation to weak tissue manipulation or suturing. Considering the pinching forces among experts in a pilot study, the maximum and mean tool–tissue forces were measured at 2.6 N (SD 0.4) and 0.9 N (SD 0.3), respectively [25]. Concerning these force levels, an accuracy of 0.4 N is insufficient for real-time force feedback on very delicate tissues.

When comparing the calibration accuracy of 0.4 N to previous sensors that were developed for tool–tissue interaction with the purpose of training and surgical safety, interesting differences were found. The force platform of Horeman et al. had a combined mean accuracy of 0.1 N despite an RMSE of 0.3 N and a $R^{2}$ of 0.99 on average per axis [25]. A force sensing robotic gripper by Zhou et al. showed a max. estimated error of around 0.5 N and an average accuracy of 0.24 N [26]. Despite these lower accuracies, all these systems seem to be accurate enough to determine the most relevant differences in force profiles related to expert levels or changes in tissue consistency.

In the SSD, StallGuard is implemented to determine the range of motion of the instrument’s tip. During the calibration sequence, both the bending range of motion and the open/close range of motion of the end-effector are determined. After calibration, users can bend, open, and close the end-effector within the safe limits of the SATA instrument’s physical endpoints. StallGuard data obtained during a calibration cycle are shown in Figure 8. StallGuard data are also monitored during end-effector closing. The tip can be actuated within its range of motion, but motor actuation is stopped once the StallGuard readings reach a preset threshold level, limiting the force the end-effector can exert. Although the back EMF force test demonstrated promising results, some technical challenges should be addressed.

According to Equation (5), back EMF measurements are proportional to motor speed. Consequently, StallGuard values are reliable only when the motors operate at a certain speed. Initially, the motors must reach this speed, making the first values not useful, as illustrated in the first 0.10 s in Figure 8. To address this issue, the initial StallGuard recordings are excluded from processing to prevent incorrect triggering of the StallGuard threshold.

Figure 8 shows that the StallGuard data exhibit strong fluctuations. Averaging these values yields more useful data (represented by the red, yellow, and purple lines in Figure 8), though it requires several data points (currently set at ten measurements, which span 0.1 s). Due to the unreliable initialisation phase and the need for averaging, there is a total measurement gap of 0.2 s at the start of each actuation. This gap limits the use of StallGuard for real-time actuation monitoring.

Furthermore, during the back EMF force tests, the averaged StallGuard values sometimes exceeded the threshold value (overshoot), after which motor actuation ceased. For example, the averaged StallGuard value obtained at one instance could be just above the threshold level, whereas the subsequent averaged StallGuard value obtained could be well below the threshold level. Consistent back EMF measurements would eliminate the need for averaging, allowing for faster and more accurate responses. This would potentially improve the accuracy of (force) features using StallGuard.

Stepper motors with a smaller mechanical step angle (angle between coils) are assumed to provide a more stable back EMF and thus StallGuard response. A test with a NEMA 18 stepper motor, with a mechanical step angle of 1.8 degrees, provided stable StallGuard data, eliminating the need for averaging. However, the bipolar stepper motors in the SSD (FIT0503) feature a mechanical step angle of 18 degrees. Furthermore, incorporating gearboxes with a ratio of 100:1 introduces friction and noise, contributing to the observed fluctuations in the obtained StallGuard data. Replacing the motors with those of higher quality with smaller mechanical step angles should improve the StallGuard results.

Moreover, StallGuard exhibits reduced effectiveness at low speeds. This is due to two reasons: firstly, at lower speeds, the coils are exposed to a lower change of magnetic flux hence less back EMF is generated (as can be derived from Equation (5)), making it harder to distinguish a shift in measurements upon motor load or stall. Secondly, at lower speeds, the rotor tends to execute more discrete steps rather than a smooth, continuous rotation. These discrete steps result in instances where speed becomes (almost) zero, thus the back EMF generated diminishes.

**Figure 12:**
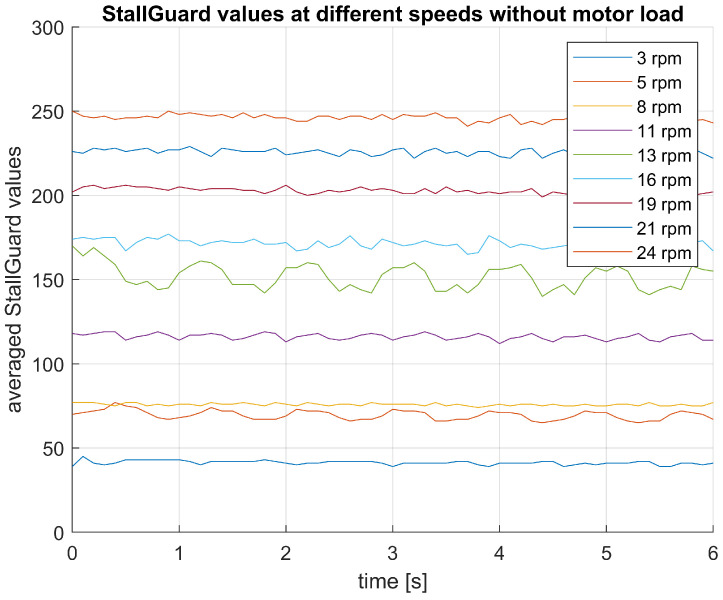
Graph of the average StallGuard values obtained from the stepper driver during unobstructed motor actuation at different rotational velocities between 3 and 24 rpm. At motor speeds lower than 11 rpm, the StallGuard value becomes too low to determine the motor load.

Therefore, it becomes challenging to distinguish normal motor operation from motor stalls or increased motor load at low motor speed. Based on the StallGuard data obtained for various speed levels during unloaded motor actuation, the motors (with 100:1 gearbox) in the SSD have to run at a minimum speed of 11 rpm to be able to properly estimate motor loading using StallGuard technology (Figure 12). Lower motor speeds generate insufficient back EMF to be able to determine motor load using StallGuard.

On the contrary, at higher speeds, the rotor moves more smoothly, and the magnetic flux ϕ changes at a higher rate, resulting in a higher back EMF (as per Equations (1) and (5)). However, measuring StallGuard at very high speeds also presents challenges. The intervals of zero-current crossings become shorter, making it difficult to measure the back EMF accurately. An inactive coil first undergoes a transient period during which the voltage drops from the supplied voltage to the back EMF voltage. If the speed increases too much, the zero-current crossing period becomes too short for the voltage to drop, complicating the back EMF measurement [27]. Additional testing is required to establish the speed levels for useful back EMF monitoring using StallGuard.

Finally, it should be noted that StallGuard measurements vary depending on the individual motor and its characteristics, as well as current and speed settings. For accurate and reliable performance, it is required to conduct baseline calibration and measurements for each motor in every application.

### 4.2. Future Work

Better stepper motors with less backlash, built-in encoders, and smaller step angles could enhance motor performance and control and might provide better data. Despite its limitations, embedded back EMF measurements are a low-budget method for detecting motor stalls, determining motor load, and estimating tool–tissue forces. This technology is a promising, cost-effective, and smart sensing solution for calibration and safety features (e.g., force feedback and force limitation). It requires no additional sensors or electronics, as it utilises already implemented components. The respective calibration and end-effector force limitation features are implemented in the current operational version of the Adlap SSD.

Further research will address enhancing the back EMF system. This model is based on measurements taken with the instrument in a straight position only. In order to develop functions that are based on real-time force feedback in the instrument end-effector, thorough characterisation of the instrument under different operating conditions is needed to map out the process for back EMF readings in the full range of instrument speeds and positions. With this data, together with the previously mentioned improvements in motors and built-in encoders, a model could be developed that provides qualitative real-time (end-effector) force feedback (e.g., visually) to operators during instrument handling and tool–tissue interactions in surgery. Another potential application of back EMF measurements is instrument identification. By configuring different ranges of motion for each type of SATA instrument or end-effector, the driver could be able to distinguish different types of instruments. This would enable the system to automatically adapt to the specific instrument being used. Back EMF measurements could also be used to detect wear and tear over time. By collecting back EMF measurements, trends and changes can be observed that might indicate wear. This also highlights a limitation of indirectly measuring the applied forces, namely that the force measured is influenced by the friction in the components between the motor and instrument. Any changes in the internal friction between components due to fluids or wear and tear result in incorrect force values. To overcome the influence on the precision of potential variability between stepper motors and noise from gearboxes, motors can be tested and selected based on performance or the friction between the gears can be reduced by adding lubrication or by using materials that have a low friction coefficient.

Lastly, future research will compare the cost, feasibility, and performance of using the back EMF system to other methods of measuring forces (monitoring motor current draw, torsional load cells, etc.). It should be considered that although the proposed sensing technology eliminates the need for extra sensors in the instrument shaft and tip, the method is not without costs due to the current sensing electronics that are needed for each motor in order to make a fair comparison between sensing technologies in the future.

## 5. Conclusions

A smart force sensing methodology based on the back electromotive force was successfully integrated into the Smart SATA Driver. Embedded back EMF-measuring technology enables motor load and motor stall detection and eliminates the need for any sensors or electronics in laparoscopic instruments, making this technology compatible with CSSD processes. This smart sensing technology was used to calibrate the instrument automatically and to provide relevant tip force data.

Further research will address enhancing the back EMF force model by using improved components and determining the influence of the instrument tip angle and various motor speeds.

Force sensing based on back EMF technology is a promising low-budget method for estimating the tool–tissue forces based on motor loading without the need for additional force sensors inside the laparoscopic instrument.

When technology is created that reduces either the complexity, the number of parts, or the maintenance costs of life-saving medical technology, implementation in less wealthy parts of the world becomes easier. When following this approach, it remains important to maintain a holistic view of health technology development (e.g., robotic systems) for under-resourced regions.

## Figures and Tables

**Table 1:** Results of the tip sensor calibration using the second exponential fit analysis.

$f\left(x\right)=a\cdot e^{\mathrm{bx}}+c\cdot e^{\mathrm{dx}}$		
Coefficient	Value	95%-CI
a	0.2549	(0.04965, 0.4601)
b	0.7279	(0.3753, 1.08)
c	3.704 × $10^{-5}$	(1.272 × $10^{-5}$, 6.136 × $10^{-5}$)
d	3.929	(3.751, 4.107)

**Table 2:** For each StallGuard threshold level, the test was conducted ten times, resulting in 110 test repetitions, divided over eleven different TCs. Once the StallGuard value reached the threshold value set (230—stall sensitivity high; 130—stall sensitivity low), motor actuation was ceased. The average tip force was measured for each TC.

Test Condition	StallGuard Threshold	Average Force [N]
TC1	230	8.2 (SD 0.4)
TC2	220	8.0 (SD 0.3)
TC3	210	7.7 (SD 0.4)
TC4	200	7.4 (SD 0.6)
TC5	190	6.6 (SD 0.3)
TC6	180	6.2 (SD 0.5)
TC7	170	5.9 (SD 0.3)
TC8	160	5.3 (SD 0.3)
TC9	150	4.6 (SD 0.4)
TC10	140	3.6 (SD 0.6)
TC11	130	2.4 (SD 0.3)

**Table 3:** Results of the regression analysis using second-order power series model.

$f\left(x\right)=a\cdot x^{b}+c$		
Coefficient	Value	95%-CI
a	−2.1 $\times \;10^{-8}$	(−9.5 $\times \;10^{-8}$, 5.3 $\times \;10^{-8}$)
b	3.6	(3.0, 4.2)
c	9.1	(8.6, 9.6)

## Data Availability

Data are contained within the article and Supplementary Materials and is openly available at our 4TU research-data repository with https://doi.org/10.4121/5156d8f6-4a7e-4145-a516-78a8a3830af9.v1.

## Supplementary Materials

The following supporting information can be downloaded at: https://www.mdpi.com/article/10.3390/s25030777/s1, Table S1: Results.

## Abbreviations

The following abbreviations are used in this manuscript:

RAS	Robot-assisted surgery
AdLap RS	Advanced laparoscopic robotic system
SATA	Shaft-actuated tip-articulating
DOF	Degree of freedom
MILSI drive	Modular interfaced sterilisable laparoscopic instrument drive
CSSD	Central services and sterilisation department
Back EMF	Back electromotive force
SSD	Smart SATA Driver
rpm	Rotations per minute
