# Supplementary material for: Smart Force Sensing in Robot Surgery Utilising the Back Electromotive Force
Source: Sensors (Basel). 2025 Jan 28;25(3):777. doi: 10.3390/s25030777 (PMC11820894; doi:10.3390/s25030777)
Supplement: Supplementary file 1 [file sensors-25-00777-s001.zip › sensors-3316230-supplementary.pdf]

Tip calibration experiment		value
Data fit	2nd exponential fit component:	
	A	0.25
	B	0.72
	C	3.7 e-5
	D	0.93
	Route Square	0.95
	RSME	0.4 Newton
Performance	Accuracy	0.5 Newton
	Range (mean accuracy <0.5N)	0-10 Newton
	Max force	35 Newton
	Sensitivity	<1.2 Newton
